# Triple and quadruple cervical artery dissections: a systematic review of individual patient data

**DOI:** 10.1007/s00415-019-09269-1

**Published:** 2019-03-23

**Authors:** Valeria Guglielmi, Jeldican Visser, Marcel Arnold, Hakan Sarikaya, René van den Berg, Paul J. Nederkoorn, Didier Leys, David Calvet, Manja Kloss, Alessandro Pezzini, Turgut Tatlisumak, Sabrina Schilling, Stéphanie Debette, Jonathan M. Coutinho

**Affiliations:** 1 Department of Neurology, Amsterdam UMC, Location AMC, Meibergdreef 9, 1105 AZ Amsterdam, The Netherlands; 2 Department of Neurology, Onze Lieve Vrouwe Gasthuis, Oosterpark 9, 1091 AC Amsterdam, The Netherlands; 3 Department of Neurology, INSELSPITAL, University Hospital Bern, Freiburgstrasse, 3010 Bern, Switzerland; 4 Department of Radiology and Nuclear Medicine, Amsterdam UMC, Location AMC, Meibergdreef 9, 1105 AZ Amsterdam, The Netherlands; 5 Department of Neurology, Lille University Hospital, Inserm U1171, Lille, France; 6 Department of Neurology, Université Paris Descartes, Centre Hospitalier Sainte Anne, Inserm U919, Paris, France; 7 Department of Neurology, Heidelberg University Hospital, Heidelberg, Germany; 8 Department of Neurology, University of Brescia, Brescia, Italy; 9 Department of Clinical Neuroscience, Institute of Neuroscience and Physiology, Sahlgrenska Academy at University of Gothenburg, Gothenburg, Sweden; 10 Department of Neurology, Sahlgrenska University Hospital, Gothenburg, Sweden; 11 University of Bordeaux, Bordeaux Population Health Research Center, Inserm Center U1219, Bordeaux, France; 12 Department of Neurology, Bordeaux University Hospital, Inserm U1219, Bordeaux, France

**Keywords:** Dissection, Cervical artery, Ischemic stroke, Cerebral infarction, Stroke

## Abstract

**Background and purpose:**

Simultaneous dissection of three or four cervical arteries rarely occurs. As a result, limited information is available on clinical characteristics, underlying causes, treatment, and outcome of these patients.

**Methods:**

We performed a systematic review of individual patient data on triple and quadruple cervical artery dissection (CeAD). We included all cases for whom, at minimum, data on age, sex and affected cervical arteries were available.

**Results:**

Out of 1396 publications identified in the initial search, 52 were included, with data available on 96 patients. Mean age was 42 years and 66% were women. 63% had triple CeAD. The most common manifestations were headache (69%), neck pain (44%), motor deficit (36%), and Horner syndrome (34%). 57% had an ischemic stroke, in the majority of these patients the stroke was confined to the vascular territory of a single artery. 83% were managed medically (antiplatelets or anticoagulants) and 11% underwent endovascular treatment. An underlying disease or triggering event was identified in 71%, most commonly trauma (35%, cervical manipulative therapy in 13%), infection (18%), fibromuscular dysplasia (16%), and hereditary connective tissue disorder (8%). In-hospital mortality was 1%. 80% of patients had a good functional outcome (mRS 0–1) at follow-up. Two recurrences (3%) were reported.

**Conclusions:**

Triple or quadruple CeAD mostly affected young women, and underlying disease or triggering event could be identified in more than two-thirds of patients. Less than two-thirds of triple or quadruple CeAD patients suffered ischemic stroke. Most patients were managed medically and the majority had a favorable outcome.

**Electronic supplementary material:**

The online version of this article (10.1007/s00415-019-09269-1) contains supplementary material, which is available to authorized users.

## Introduction

Cervical artery dissection (CeAD) accounts for approximately 20% of strokes in patients less than 45 years of age [1]. In most cases, dissection occurs in a single artery. Simultaneous dissection of three or four cervical arteries is a rare occurrence. About 2–4% of all CeAD comprise of triple or quadruple dissections, compared to 13–23% double and 73–85% single dissections [2–5]. There are a few case reports or case series on triple or quadruple dissections and large cohort studies generally only report in terms of single or multiple dissections, without distinguishing patients within the latter group. Because of the paucity of data on triple and quadruple CeAD, little is known about the clinical manifestations, underlying causes, treatment, and outcome of these patients. The aim of the current study was to perform a systematic review of individual patient data on triple and quadruple CeAD.

## Patients and methods

We performed a systematic review according to Preferred Reporting Items for Systematic Reviews and Meta-Analyses of Individual Patient Data (PRISMA-IPD) guidelines [6]. We searched Pubmed and Excerpta Medica Database (EMBASE) (from inception to April 17, 2018) using a predefined search strategy including the terms multiple, triple, or quadruple cervical artery dissection (Online Resource Fig. I). Full-length articles and conference abstracts were reviewed. The primary search was performed independently by two authors (V.G. and J.M.C). Full-length articles of potentially relevant studies were reviewed by one of the authors (V.G). Triple or quadruple CeAD was considered simultaneous if the dissections were simultaneously present on baseline imaging of the cervical arteries, or if on baseline imaging an initial dissection was visible and additional dissections were diagnosed within 30 days. For inclusion in the study, triple or quadruple CeAD had to have been diagnosed by MRI, MRA, CT-angiography (CTA), catheter angiography, or autopsy, on the basis of at least one of the following criteria: (1) double lumen; (2) mural hematoma; (3) tapering occlusion or long tapering stenosis. We included all cases for which at minimum the following individual patient data were available: age, sex, and affected cervical arteries. If this information could not be extracted from the article, the corresponding author was contacted to provide relevant data on five separate occasions. Articles written in languages other than English, French, German, Spanish, Italian or Dutch were included if they had an English abstract that contained sufficient data. Fibromuscular dysplasia (FMD) diagnosis had to be based on histological examination or multifocal arterial stenosis with ‘string-of-beads’ appearance on catheter angiography, MRA, or CTA. Hereditary connective tissue disorders had to be confirmed by genetic testing. Trauma was defined as any reported mechanical triggering event with impact on the head or neck, including major or minor trauma (e.g. motor vehicle accident, fall, notable head/neck movements, sports activities, cervical manipulative therapy). Medical management was defined as treatment with antiplatelets or anticoagulants, without surgery or endovascular intervention. As main clinical outcome, we used the modified Rankin Scale (mRS). If outcome was only reported descriptively, we attempted to construct a mRS score from this information (e.g. ‘full recovery’—mRS 0). Recanalization at follow-up imaging was defined as improved arterial flow (partial or complete recanalization) in all affected arteries. If one or more arteries remained occluded, recanalization was reported as absent. Data were summarized using descriptive statistics (medians, means, SDs, frequencies), using IBM SPSS Statistics V.23 (IBM). We also compared key findings of the current study to available data of patients with double and single CeAD from the Cervical Artery Dissection and Ischemic Stroke Patients (CADISP) study, one of the largest multicenter cohort studies in the literature [3]. Data of patients with triple/quadruple vs single CeAD were analyzed using the OpenEpi^®^ software program (Open Source Epidemiologic Statistics for Public Health V3.01). For comparison of continuous data, we used a two-sided independent *t* test and for a comparison of proportions, we used a chi-square test or Fisher’s exact test, whichever was appropriate.

## Results

A flowchart of study selection is provided in Online Resource Fig. I. Of the 1396 manuscripts identified in the initial search, 168 were selected for full-length review. Of these, 52 studies were included, containing individual patient data of 96 patients. Study characteristics are depicted in Online Resource Table I. The studies were published between 1977 and 2017. 41 studies were case reports or case series. Clinical characteristics are reported in Table 1. 8% of patients presented with only headache or neck pain without additional neurological symptoms.

**Table 1:** Clinical characteristics

	*n*/*N* (%)
Demographics	
Mean age, year (SD)^a^	42 (9)
Sex (% women)	63/96 (66%)
Medical history	
Current or past smoking	20/72 (28%)
Hypertension	16/72 (22%)
Migraine	25/72 (35%)
Diabetes	4/72 (6%)
Hypercholesterolemia	12/72 (17%)
Oral contraceptive use (women)	6/63 (14%)
Clinical characteristics	
Headache	63/92 (69%)
Neck pain	40/92 (44%)
Headache or neck pain as the only symptom	8/96 (8%)
Motor deficit	26/72 (36%)
Horner syndrome	31/92 (34%)
TIA	17/92 (19%)
Median duration symptom onset, diagnosis CeAD (range)^b^	4 days (0–31)

*CeAD* cervical artery dissection

^a^Calculated from data of 96 patients

^b^Calculated from data of 46 patients

MRI was performed in 83% of patients, MRA in 67%, catheter angiography in 59%, CTA in 23%, and autopsy in 1%. In 88/96 (92%) patients, triple or quadruple CeAD were simultaneously identified on baseline imaging. In 8/96 (12%) patients, an initial dissection was visible on baseline imaging, followed by diagnosis of additional dissections within a median of 4 days (IQR 1–17). Data on radiological findings, treatment and outcome are presented in Table 2. 63% had triple and 37% had quadruple CeAD. 57% of patients had an ischemic stroke at presentation or during hospitalization. Information on the duration of antithrombotic treatment was available for 64% of the patients who were managed medically. 4% were treated for less than 6 months, 58% for (at least) 6 months, 36% long term, but without further specification, and 2% indefinitely. 11% underwent endovascular stent placement. The median time between symptom onset and the endovascular procedure was 3 days (range 1–120). In half of these cases, the reason for the intervention was neurologic deterioration despite medical management. One patient (1%) died during hospitalization as a result of hemorrhagic transformation of a cerebral infarct. This patient was a 49-year-old man with fibromuscular dysplasia and quadruple CeAD which had occurred during skiing. Two recurrent CeADs were reported.

**Table 2:** Radiological findings, treatment, and outcome

	*n*/*N* (%)
Radiological findings	
Triple CeAD	60/96 (63%)
2xICA and 1xVA	40/60 (67%)
Ischemic stroke	52/91 (57%)
1 cervical artery vascular territory	23/36 (64%)
Subarachnoid hemorrhage	6/90 (7%)
Intracerebral hemorrhage^a^	5/90 (6%)
Treatment	
Medical management	70/84 (83%)
Antiplatelets	17/84 (20%)
Anticoagulants	23/84 (27%)
Antiplatelets and anticoagulants^b^	28/84 (33%)
Unspecified	2/84 (2%)
Endovascular stent placement	9/84 (11%)
Surgery	1/84 (1%)
None of the above	4/84 (5%)
mRS score at last clinical follow-up^c^	
0	39/74 (53%)
1	20/74 (27%)
2	6/74 (8%)
3	4/74 (5%)
4	4/74 (5%)
5	0/74 (0%)
6	1/74 (1%)
Recurrence of CeAD	2/80 (3%)
Recanalization^d^	28/31 (90%)

*CeAD* cervical artery dissection, *ICA* internal carotid artery, *mRS* modified Rankin Scale, *VA* vertebral artery

^a^Hemorrhagic transformation of a cerebral infarct in 4 patients, related to traumatic injuries in 1 patient

^b^4 patients received both concurrently; 22 sequentially; for 2 the order of administration was not provided, respectively

^c^In 55% a documented mRS was available, in 45% it was imputed. Median time to last clinical follow-up (range): 4 months (0–98). Outcome (mRS) in patients with ischemic stroke was: 0, 35%; 1, 33%; 2, 13%; 3, 8%; 4, 10%; 5, 0%; 6; 3%. Outcome (mRS) in patients without ischemic stroke was: 0, 72%; 1, 22%; 2, 3%; 3, 3%; 4–6, 0%

^d^Last imaging follow-up: <3 months in 5 patients; 3–6 months in 16; 6–12 months in 5; >12 months in 3; and not specified for 2

Information on underlying causes of CeAD was available for 94/96 patients and in 67 (71%) an underlying disease or triggering event could be identified (Table 3). 12 patients (eight women) were diagnosed with FMD. Three patients (two women) had Ehlers–Danlos type IV and one woman had osteogenesis imperfecta type I. Triggering events included infection (*n* = 16), trauma (*n* = 32, cervical manipulative therapy in 12/32 patients), recent childbirth (*n* = 5), and other (*n* = 17).

**Table 3:** Underlying disease and/or triggering events

	*n*/*N* (%)
Underlying disease and or triggering event identified	67/94 (71%)
Fibromuscular dysplasia^a^	12/74 (16%)
Hereditary connective tissue disorder^b^	4/50 (8%)
Recent infection^c^	16/90 (18%)
Trauma^d^	32/90 (35%)
Cervical manipulative therapy	12/90 (13%)
Recent childbirth (women)^e^	5/59 (7%)
Other^f^	17/94 (18%)
None identified	27/94 (29%)
Unknown	2/96 (2%)
Median duration risk factor, symptom onset (range)^g^	5 days (0–22)

In 9 patients both an underlying disease and a triggering event were identified. In 9 other patients more than 1 triggering event was identified

^a^8 women, 4 men

^b^Ehlers–Danlos type IV in 3 patients (2 women) and osteogenesis imperfecta type I in 1 (woman)

^c^Respiratory in 10, gastro-intestinal in 2, sinusitis in 2, hepatitis C in 1, and 1 patient had elevated inflammatory parameters without an identified focus

^d^Motor vehicle accident in 8, fall from skiing in 1, fall from a horse in 1, fall in 1; heavy load carrying in 1, head extension in 2, and other head movements in 2; cervical manipulative therapy in 12; sports activities in 6: skiing in 1, scuba diving in 1, river rafting in 1, cycling in 1, yoga in 1, heavy weight lifting in 1

^e^Cesarean section in 4 and vaginal delivery in 1 woman

^f^Other factors that were considered triggering events by authors of included publications: recent head/neck surgery in 1, head/neck surgery in distant medical history in 9, reversible vasoconstriction syndrome in 3, chronic ergotism in 1, alemtuzumab therapy in 1, iatrogenic vessel wall damage during angiography in 1, positive thrombophilic markers in 1 patient

^g^Calculated from data of 27 patients. The nine patients with head/neck surgery in distant medical history were excluded (median duration risk factor, symptom onset was 19 years; range 9–50 year)

An overview of key findings of the current study compared to available data of patients with double and single CeAD from the Cervical Artery Dissection and Ischemic Stroke Patients (CADISP) study [3] is provided in Online Resource Table II. Patients with triple/quadruple CeAD were more often female (66% vs 42%, *p* < 0.001), more often had underlying fibromuscular dysplasia (16% vs 4%, *p* < 0.001) or a hereditary connective tissue disorder (8% vs 0.4%, *p* < 0.001) compared to patients with single CeAD. There was no difference in clinical outcome (mRS 0–2 88% vs. 88%, *p* = 0.951) or frequency of recurrent CeAD (3% vs 2%, *p* = 1.000).

## Discussion

This is the first systematic review on triple and quadruple CeAD. Despite the rarity of this condition, we were able to collect data from almost 100 patients. In most patients a cause or risk factor for CeAD was identified, stressing the need for a careful patient history and diagnostic work-up in these patients. Most patients with triple or quadruple CeAD were managed medically. While CeAD was complicated by a stroke or TIA in the majority of patients, in-hospital mortality was low and clinical outcome was favorable (mRS 0–1) in 80% of patients.

Cohort studies of CeAD suggest that an inherited connective tissue disorder is found in less than 0.5% of patients in patients with single CeAD [7] [Online Resource Table II], which is considerably lower than the 8% in our systematic review. Similarly, FMD was found in only 4% of CADISP study patients with a single CeAD [3] [Online Resource Table II], compared to 16% in our systematic review. This would suggest that patients with triple or quadruple CeAD are more likely to have an underlying connective tissue disorder. However, these differences may also be a result of publication or verification bias, as triple or quadruple CeAD likely leads to more thorough ancillary investigation.

There is usually no sex predominance in European populations for cervical artery dissections. However, in studies reporting single and multiple CeAD separately, there does seem to be a trend towards higher proportion of women in multiple CeAD [3, 5]. The skewed sex ratio (with two-thirds of women) we observed may be explained by sex-specific risk factors such as FMD, which occurs in women in about 90% of cases, and recent childbirth.

18% of patients had a recent infection, which is similar to the frequency among patients with single CeAD [3, 5] (Online Resource Table II). This association may be due to mechanical forces such as violent coughing, sneezing or vomiting, or due to a transient inflammatory arteriopathy [8]. Recent cervical manipulative therapy was another triggering event that was frequently reported. Various controlled studies have observed an association between manipulative therapy and CeAD, particularly of the vertebral arteries [9] and multiple CeAD [3]. Despite the clear association, it remains unknown whether there is also a causal relation, since patients may have sought manipulative therapy because of neck pain caused by the dissection. At the very least, therapists should inform patients on the possible risk of causing CeAD prior to performing manipulative therapy.

Five women had recently given birth. As most of the patients in this study were women within child-bearing age range, this association may be coincidental. Other explanatory theories include mechanical pressure on vessel walls associated with protracted delivery, hyperextension of the neck during (general) anesthesia or hemodynamic and hormonal changes related to pregnancy and postpartum that may lead to transient vulnerability of the vessel walls [10].

In one-third of patients no underlying disease or triggering event was identified. Triple or quadruple vessel dissections without any reason seem unlikely. The underlying cause may be a yet to be defined genetic arteriopathy, transient (inflammatory) vulnerability of the vessel walls, mechanical factors such as minor trauma related to daily activities and sports activities, or any combination of the above.

There are no evidence-based guidelines on the optimal treatment of triple or quadruple CeAD. Our data show that most of these patients are managed medically. We did observe treatment variation in the type of medical therapy, with both antiplatelets and anticoagulants used in similar frequencies. In the CADISS trial, no difference was found between antiplatelets and anticoagulants in the prevention of stroke and death in patients with single CeAD [11].

Surprisingly, 88% of patients with triple or quadruple CeAD had no or only minor disability at follow-up (mRS 0–2), and the proportion of good outcome is comparable to the outcome in a large cohort of patients with double and single CeAD (88% mRS 0–2) [3] (Online Resource Table II). One potential explanation may be that only 57% of triple or quadruple CeAD patients suffered ischemic stroke, and that in most patients, stroke was confined to the vascular territory of a single artery. The proportion of ischemic stroke at baseline in triple or quadruple CeAD appears to be lower than reported in single CeAD (74–76%) [3, 5] and similar to double CeAD (67%) (Online Resource Table II). Perhaps because local symptoms are more pronounced in multiple dissections, the likelihood of diagnosing the condition before ischemic stroke has occurred is higher. Alternatively, selective publication of patients with triple or quadruple CeAD and a good outcome may also play a role.

Recurrences of CeAD were reported in only 3%, which is similar to a recent large cohort study of patients with double and single CeAD and comparable follow-up period [3] [Online Resource Table II]. In an earlier smaller study of 200 patients with CeAD (46 patients with double and 9 patients with triple or quadruple CeAD) and a median follow-up period of 7.4 years, recurrent CeAD occurred in 8% of patients, but only in arteries that were not previously affected [4]. The risk of recurrence was highest in the first month, and after the first month the risk of recurrent dissection was 1% per year. According to another recent study, patients with late recurrent CeAD tend to be younger than those with recurrent CeAD within the first month (mean age 37.5 ± 6.9 vs 43.8 ± 9.9) [12]. Based on our data, the risk of recurrence does not appear to be higher in patients with triple and quadruple CeAD. This low risk of recurrence in patients with triple and quadruple CeAD argues in favor of a transient arteriopathy rather than an underlying genetic arteriopathy in the majority of patients.

Our review has several limitations. First, despite our best efforts, we could not acquire individual patient data from 3 out of 55 studies. Second, there is almost certainly some degree of publication bias, which may lead to an overestimation of good clinical outcome and low recurrence rate. Third, recall bias may play a role in the large number of reported triggering events. Fourth, the absence of a reference group limits the interpretation of the findings. In an attempt to improve interpretation, we compared key findings of our study to available literature on double and single dissections from one of the largest and most well-defined CeAD multicenter cohort studies. Of course, this comparison must be interpreted with caution, since it is subject to data collection bias. Fifth, although triple and quadruple CeAD were simultaneously present on baseline imaging in the vast majority of patients in our study, we cannot be certain that they occurred at the same point in time. The distinction between multiple and early recurrent dissections is a matter of debate and depends in part on the delay between symptom onset and diagnosis by cervical artery imaging [5, 12]. Sixth, there was no centralized reading of imaging and results from the CADISS trial have shown that diagnosis of CeAD can be difficult [11].

## Conclusion

In conclusion, our systematic review on triple or quadruple dissections shows that this condition mostly affects young women, and that an underlying disease or triggering event can be identified in more than two-thirds of patients. Less than two-thirds of triple or quadruple CeAD patients suffered ischemic stroke, and the stroke was usually confined to the vascular territory of a single artery. Most patients were managed medically and the prognosis of triple and quadruple CeAD was favorable in the majority of patients, with a low recurrence risk.

## Electronic supplementary material

Below is the link to the electronic supplementary material.

Supplementary material 1 (PDF 119 KB)

Supplementary material 2 (PDF 91 KB)

Supplementary material 3 (PDF 27 KB)

Supplementary material 4 (PDF 141 KB)
