# Supplementary material for: Triple and quadruple cervical artery dissections: a systematic review of individual patient data
Source: J Neurol. 2019 Mar 23;266(6):1383–8. doi: 10.1007/s00415-019-09269-1 (PMC6517349; doi:10.1007/s00415-019-09269-1)
Supplement: Supplementary file 1 — Supplementary material 1 (PDF 119 KB) [file 415_2019_9269_MOESM1_ESM.pdf]

Online Resource

FIgure I PRISMA IPD Flow Diagram

Triple and quadruple cervical artery dissections: a systematic review of individual patient data
Journal of Neurology

Valeria Guglielmi, Jeldican Visser, Marcel Arnold, Hakan Sarikaya, René van den Berg, Paul J. Nederkoorn, Didier Leys, David Calvet, Manja Kloss, Vincent Thijs, Alessandro Pezzini, Turgut Tatlisumak, Sabrina Schilling, Stéphanie Debette, Jonathan M. Coutinho. Correspondence to: Jonathan M. Coutinho, Department of Neurology, Amsterdam UMC, location AMC, Amsterdam, The Netherlands. E-mail: j.coutinho@amc.nl

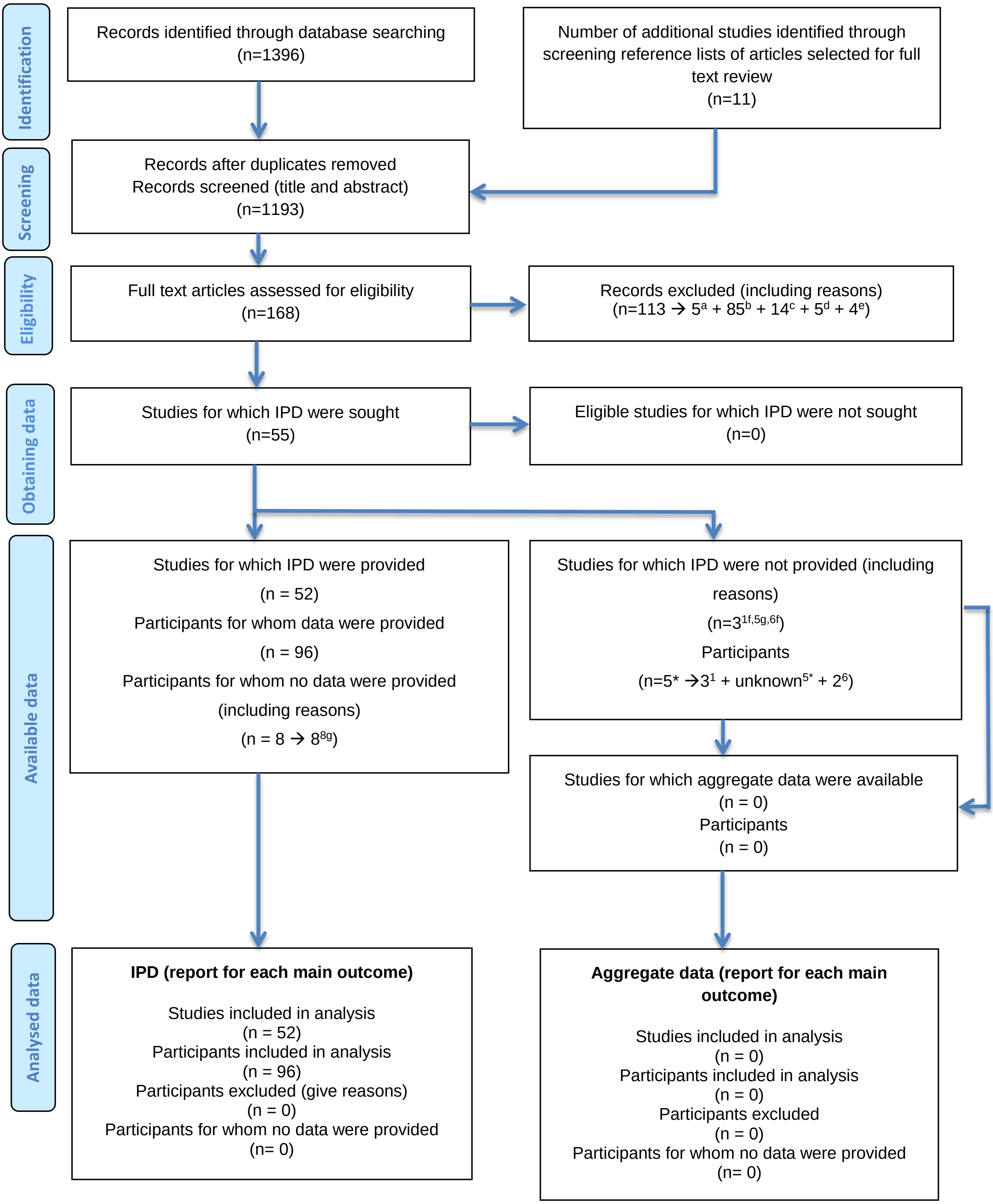

The PRISMA IPD flow diagram

© Reproduced with permission of the PRISMA IPD Group, which encourages sharing and reuse for non commercial purposes

Search strategy

We searched PubMed and Excerpta Medica Database (EMBASE) (until April 17, 2018) for publications on triple and quadruple CeAD using the terms. We used the following search terms: ((("cervical artery" OR "cervical arteries" OR "cervical arterial" OR "cervical vertebrae" OR "vertebral artery" OR "vertebral arteries" OR "vertebral arterial" OR "carotid artery" OR "carotid arteries" OR "carotid arterial" OR "cervicocerebral artery" OR "cervicocerebral arteries" OR "cervicocerebral arterial" OR "cervicocephalic artery" OR "cervicocephalic arteries" OR "cervicocephalic arterial") AND (dissection OR dissecting)) AND (fourfold OR quadruple OR triple OR multiple).

Reasons for exclusion

[a] Full text article was written in a language other than English, French, German, Spanish, Italian or Dutch and the English abstract had insufficient data for inclusion.

[b] Not about triple or quadruple cervical artery dissection.

[c] Does not meet imaging criteria for diagnosis of triple or quadruple article dissection (at least one of the following: 1) double lumen, 2) mural hematoma, 3) 'string sign' or 'flame sign': long tapering occlusion or high grade stenosis).

[d] Duplicate study.

[e] Duplicate participants.

[f] The first and corresponding authors did not respond to five e-mails spanning a 7 month period, requesting data.

[g] The corresponding author no longer had data available for these participants.

[*] In one study it was not explicitly stated how many participants had triple or quadruple cervical artery dissections.

References of studies for which individual patient data were requested from corresponding authors

1. Baracchini C, Tonello S, Vitaliani R, Giometto B, Meneghetti G, Ballotta E. Vasomotion in multiple spontaneous cervical artery dissections. *Stroke*. 2008;39:1148-1151.
2. Debette S, Grond-Ginsbach C, Bodenant M, Kloss M, Engelter S, Metso T, et al. Differential features of carotid and vertebral artery dissections: the CADISP study. *Neurology*. 2011;77:1174-1181.
3. Guillon B, Berthet K, Benslamia L, Bertrand M, Bousser MG, Tzourio C. Infection and the risk of spontaneous cervical artery dissection: A case-control study. *Stroke; a journal of cerebral circulation*. 2003;34:e79-81.
4. Kloss M, Wiest T, Hyrenbach S, Werner I, Arnold ML, Lichy C, et al. Mthfr 677tt genotype increases the risk for cervical artery dissections. *Journal of Neurology, Neurosurgery and Psychiatry*. 2006;77:951-952.
5. Mokri B, Houser OW, Sandok BA, Piepgras DG. Spontaneous dissections of the vertebral arteries. *Neurology*. 1988;38:880-885.
6. Pelkonen O, Tikkakoski T, Leinonen S, Pyhtinen J, Lepojarvi M, Sotaniemi K. Extracranial internal carotid and vertebral artery dissections: Angiographic spectrum, course and prognosis. *Neuroradiology*. 2003;45:71-77.
7. Pfefferkorn T, Saam T, Rominger A, Habs M, Gerdes LA, Schmidt C, et al. Vessel wall inflammation in spontaneous cervical artery dissection: A prospective, observational positron emission tomography, computed tomography, and magnetic resonance imaging study. *Stroke*. 2011;42:1563-1568.
8. Schievink WI, Mokri B, O'Fallon WM. Recurrent spontaneous cervical-artery dissection. *New England Journal of Medicine*. 1994;330:393-397.
9. Schwartz NE, Vertinsky AT, Hirsch KG, Albers GW. Clinical and radiographic natural history of cervical artery dissections. *J Stroke Cerebrovasc Dis*. 2009;18:416-423.
