# Supplementary material for: Triple and quadruple cervical artery dissections: a systematic review of individual patient data
Source: J Neurol. 2019 Mar 23;266(6):1383–8. doi: 10.1007/s00415-019-09269-1 (PMC6517349; doi:10.1007/s00415-019-09269-1)
Supplement: Supplementary file 2 — Supplementary material 2 (PDF 91 KB) [file 415_2019_9269_MOESM2_ESM.pdf]

Online Resource

Triple and quadruple cervical artery dissections: a systematic review of individual patient data
Journal of Neurology

Valeria Guglielmi, Jeldican Visser, Marcel Arnold, Hakan Sarikaya, René van den Berg, Paul J. Nederkoorn, Didier Leys, David Calvet, Manja Kloss, Alessandro Pezzini, Turgut Tatlisumak, Sabrina Schilling, Stéphanie Debette, Jonathan M. Coutinho
Correspondence to: Jonathan M. Coutinho, Department of Neurology, Amsterdam UMC, location AMC, Amsterdam, The Netherlands. E-mail: j.coutinho@amc.nl

Table I Study Characteristics of included studies

First author	Year of publication	Type of study*	Retrospective or prospective	Number of patients†, (included)
Ringel[1]	1977	Case report	Retrospective	1 (1)
Mas[2]	1985	Case series, unclear whether single or multicenter	Retrospective	2 (2)
Schievink[3]‡	1994	Cohort, single center	Unclear	9 (1)
Mayer[4]	1996	Case report	Retrospective	1 (1)
Grau[5]	1997	Case series, unclear whether single or multicenter	Retrospective	1 (1)
Rees[6]	1997	Case report	Retrospective	1 (1)
Coric[7]	1998	Case report	Retrospective	1 (1)
Eachempati[8]	1998	Case report	Retrospective	1 (1)
Wunderlich[9]	1999	Case report	Retrospective	1 (1)
Busch[10]	2000	Case report	Retrospective	1 (1)
Garnier[11]	2000	Case report	Retrospective	1 (1)
Buyle[12]	2001	Case report	Retrospective	1 (1)

Guillon[13]	2003	Case-control, multicenter	Prospective	2 (2)
Hamann[14]	2003	Case report	Retrospective	1 (1)
Oehler[15]	2003	Case report	Retrospective	1 (1)
Nadgir[16]	2003	Case report	Retrospective	1 (1)
Pace[17]	2004	Case series, single center	Retrospective	1 (1)
Yong[18]	2005	Case report	Retrospective	1 (1)
Arnold[19]	2006	Cohort, single center	Prospective	1 (1)
Kloss[20]§	2006	Cohort, single center	Unclear	7 (7)
Molina-Martinez[21]	2006	Case report	Retrospective	1 (1)
Dittrich[22]	2007	Cohort, single center	Prospective	1 (1)
Marshman[23]	2007	Case report	Retrospective	1 (1)
Tayal[24]	2007	Case report	Retrospective	1 (1)
Chakrapani[25]	2008	Case report	Retrospective	1 (1)
Lee[26]	2008	Case report	Retrospective	1 (1)
Verdure[27]	2008	Case series, single center	Retrospective	1 (1)
Arnold[28]	2009	Cohort, multi center	Prospective	12 (12)
Cerutti[29]	2009	Case report	Retrospective	1 (1)
Schwartz[30]§§	2009	Cohort, single center	Retrospective	7 (6)
Dienstknecht[31]	2010	Case report	Retrospective	1 (1)
Smith[32]	2010	Case series, single center	Retrospective	1 (1)

Albuquerque[33]	2011	Cohort, single center	Retrospective	1 (1)
Debette[34]	2011	Cohort	Retrospective and prospective	19 (19)
Papagiannaki[35]	2011	Case report	Retrospective	1 (1)
Pfefferkorn[36]	2011	Cohort, single center	Prospective	2 (2)
Pires[37]	2011	Case report	Retrospective	1 (1)
Adamec[38]	2012	Case report	Retrospective	1 (1)
Dohle[39]	2012	Case report	Retrospective	1 (1)
Grond-Ginsbach[40]	2012	Case series, multicenter	Unclear	1 (1)
Morren[41]	2012	Case report	Retrospective	1 (1)
Allam[42]	2013	Case report	Retrospective	1 (1)
Mawet[43]	2013	Cohort, single center	Prospective	2 (2)
Panicio[44]	2013	Case report	Retrospective	1 (1)
Aronov[45]	2014	Case report	Retrospective	1 (1)
Goyal[46]	2014	Case report	Retrospective	1 (1)
Vela-Duarte[47]	2014	Case report	Retrospective	1 (1)
Durand-Dubief[48]	2015	Case report	Retrospective	1 (1)
Melikyan[49]	2015	Case report	Retrospective	1 (1)
Abe[50]	2016	Case report	Retrospective	1 (1)
Vannucchi[51]	2016	Case report	Retrospective	1 (1)
Akamatsu[52]	2017	Case series, unclear whether single or multicenter	Retrospective	1 (1)

* Case report was defined as 1 patient; Case series, ≤ 10 patients; Cohort, >10 patients.

† Only the number of patients with triple or quadruple cervical artery dissection is provided, () indicates which of these patients were included in the current study. The original publication may describe additional patients.

‡ The corresponding author no longer had data available for the remaining 8 patients for which there was insufficient individual patient data available in the publication.
§ In the original publication 14 patients with triple or quadruple cervical artery dissections were reported. However, the corresponding author explained that only 8 presented with simultaneous triple or quadruple cervical artery dissections. One of these 8 patients was already described in the case report by Oehler et al (2003) and was therefore excluded.

§§ In the original publication 7 patients with triple or quadruple cervical artery dissections were reported. However, the corresponding author explained that upon further review there were only 6 patients, not 7 as stated in the paper.

References of included studies

1. Ringel SP, Harrison SH, Norenberg MD, Austin JH. Fibromuscular dysplasia: Multiple "spontaneous" dissecting aneurysms of the major cervical arteries. *Annals of neurology*. 1977;1:301-304
2. Mas JL, Goeau C, Bousser MG, Chiras J, Verret JM, Touboul PJ. Spontaneous dissecting aneurysms of the internal carotid and vertebral arteries--two case reports. *Stroke*. 1985;16:125-129
3. Schievink WI, Mokri B, O'Fallon WM. Recurrent spontaneous cervical-artery dissection. *New England Journal of Medicine*. 1994;330:393-397
4. Mayer SA, Rubin BS, Starman BJ, Byers PH. Spontaneous multivessel cervical artery dissection in a patient with a substitution of alanine for glycine (g13a) in the alpha1(i) chain of type i collagen. *Neurology*. 1996;47:552-556
5. Grau AJ, Brandt T, Forsting M, Winter R, Hacke W. Infection-associated cervical artery dissection. Three cases. *Stroke*. 1997;28:453-455
6. Rees JH, Valentine AR, Llewelyn JG. Spontaneous bilateral carotid and vertebral artery dissection presenting as a collet-sicard syndrome. *Br J Radiol*. 1997;70:856-858
7. Coric D, Wilson JA, Regan JD, Bell DA. Primary stenting of the extracranial internal carotid artery in a patient with multiple cervical dissections: Technical case report. *Neurosurgery*. 1998;43:956-959
8. Eachempati SR, Sebastian MW, Reed RL, 2nd. Posttraumatic bilateral carotid artery and right vertebral artery dissections in a patient with fibromuscular dysplasia: Case report and review of the literature. *J Trauma*. 1998;44:406-409
9. Wunderlic G, Neumann-Haefelin T, Aulich A, Steinmetz H. Triple spontaneous cervical artery dissection. *Neuroradiology*. 1999;41:433-435
10. Busch T, Aleksic I, Sirbu H, Kersten J, Dalichau H. Complex traumatic dissection of right vertebral and bilateral carotid arteries: A case report and literature review. *Cardiovasc Surg*. 2000;8:72-74
11. Garnier P, Michel D, Barral FG, Beauchet O, Ollagnier M, Guy C, et al. [roles of arterial dysplasia, chronic ergotism and other factors in a case multiple spontaneous arterial dissections]. *Rev Med Interne*. 2000;21:701-704
12. Buyle M, Engelborghs S, Kunnen J, De Deyn PP. Headache as only symptom in multiple cervical artery dissection. *Headache*. 2001;41:509-511
13. Guillon B, Berthet K, Benslamia L, Bertrand M, Bousser MG, Tzourio C. Infection and the risk of spontaneous cervical artery dissection: A case-control study. *Stroke; a journal of cerebral circulation*. 2003;34:e79-81

14. Hamann GF, Zachoval R, Von Einsiedel H, Sander D. Hepatitis-c-associated multiple arterial dissection of cerebral vessels. *European Neurology*. 2003;49:180-182
15. Oehler J, Lichy C, Gandjour J, Fiebach J, Grau AJ. [dissection of four cerebral arteries after protracted birth]. *Nervenarzt*. 2003;74:366-369
16. Nadgir RN, Loevner LA, Ahmed T, Moonis G, Chalela J, Slawek K, et al. Simultaneous bilateral internal carotid and vertebral artery dissection following chiropractic manipulation: Case report and review of the literature. *Neuroradiology*. 2003;45:311-314
17. Pace F, Toni D, Di Angelantonio E, Lorenzano S, Argentino C. Spontaneous multiple cervical artery dissection: Two case reports and a review of the literature. *Journal of Emergency Medicine*. 2004;27:133-138
18. Yong RL, Heran NS. Traumatic carotid cavernous fistula with bilateral carotid artery and vertebral artery dissections. *Acta Neurochir (Wien)*. 2005;147:1109-1113; discussion 1113
19. Arnold M, Cumurciuc R, Stapf C, Favrole P, Berthet K, Bousser MG. Pain as the only symptom of cervical artery dissection. *Journal of Neurology, Neurosurgery and Psychiatry*. 2006;77:1021-1024
20. Kloss M, Wiest T, Hyrenbach S, Werner I, Arnold ML, Lichy C, et al. Mthfr 677tt genotype increases the risk for cervical artery dissections. *J Neurol Neurosurg Psychiatry*. 2006;77:951-952
21. Molina-Martinez FJ, Salinas-Inigo ME, Moreno-Rojas AJ, Miralbes-Celma S. A quadruple spontaneous dissection of cervicocerebral arteries [2]. *European Journal of Neurology*. 2006;13:94-95
22. Dittrich R, Nassenstein I, Bachmann R, Maintz D, Nabavi DG, Heindel W, et al. Polyarterial clustered recurrence of cervical artery dissection seems to be the rule. *Neurology*. 2007;69:180-186
23. Marshman LAG, Ball L, Jadun CK. Spontaneous bilateral carotid and vertebral artery dissections associated with multiple disparate intracranial aneurysms, subarachnoid hemorrhage and spontaneous resolution. Case report and literature review. *Clinical Neurology and Neurosurgery*. 2007;109:816-820
24. Tayal V, Platts J, Smith T, White R. Acute confusional state following a whiplash injury: A case of multiple cervical artery dissection [8]. *Journal of Neurology*. 2007;254:402-403
25. Chakrapani AL, Zink W, Zimmerman R, Riina H, Benitez R. Bilateral carotid and bilateral vertebral artery dissection following facial massage. *Angiology*. 2008;59:761-764
26. Lee ST, Kim JA, Jang SY, Kim DK, Kim JW, Ki CS. A novel col3a1 gene mutation in patient with aortic dissected aneurysm and cervical artery dissections. *Heart and Vessels*. 2008;23:144-148
27. Verdure P, Triquenot-Bagan A, Perdu J, Gerardin E, Laquerriere A, Hannequin D, et al. Multiple cervical arterial dissections in two brothers: Fibromuscular dysplasia or connective tissue disease?. [french]. *Revue Neurologique*. 2008;164:F211-F215

28. Arnold M, De Marchis GM, Stapf C, Baumgartner RW, Nedeltchev K, Buffon F, et al. Triple and quadruple spontaneous cervical artery dissection: Presenting characteristics and long-term outcome. *Journal of Neurology, Neurosurgery and Psychiatry*. 2009;80:171-174
29. Cerutti D, Bonafe A, Pelouze GA, Kassem Z, Filipov R, Runavot G, et al. [endovascular stent-assisted angioplasty after cervical arteries dissection]. *Rev Neurol (Paris)*. 2010;166:333-336
30. Schwartz NE, Vertinsky AT, Hirsch KG, Albers GW. Clinical and radiographic natural history of cervical artery dissections. *J Stroke Cerebrovasc Dis*. 2009;18:416-423
31. Dienstknecht T, Ernstberger A, Nerlich M, Angele P. Asymptomatic dissection of craniocervical arteries in combination with severe cervical spine fractures. *Acta Orthopaedica Belgica*. 2010;76:282-285
32. Smith MJ, Santillan A, Segal A, Patsalides A, Gobin YP. Interventional management for secondary intracranial extension of spontaneous cervical arterial dissection. *Surg Neurol Int*. 2010;1:82
33. Albuquerque FC, Hu YC, Dashti SR, Abla AA, Clark JC, Alkire B, et al. Craniocervical arterial dissections as sequelae of chiropractic manipulation: Patterns of injury and management. *J Neurosurg*. 2011;115:1197-1205
34. Debette S, Grond-Ginsbach C, Bodenant M, Kloss M, Engelter S, Metso T, et al. Differential features of carotid and vertebral artery dissections: The cadisp study. *Neurology*. 2011;77:1174-1181
35. Papagiannaki C, Cottier JP, Barbier C, Bibi R, Herbreteau D. Quadruple spontaneous cervical artery dissection following aneurysm embolization: A rare posttreatment complication. *American Journal of Neuroradiology*. 2011;32:E3-E4
36. Pfefferkorn T, Saam T, Rominger A, Habs M, Gerdes LA, Schmidt C, et al. Vessel wall inflammation in spontaneous cervical artery dissection: A prospective, observational positron emission tomography, computed tomography, and magnetic resonance imaging study. *Stroke*. 2011;42:1563-1568
37. Pires C, Geraldes R, Neto L, Melo TP. Spontaneous multiple cervical artery dissection in the puerperium. *Cerebrovascular Diseases*. 2011;32:511-512
38. Adamec I, Matijevic V, Pavlisa G, Zadro I, Habek M. Beware of "old" horner syndrome. *Optometry and vision science : official publication of the American Academy of Optometry*. 2012;89:e12-15
39. Dohle C, Baehring JM. Multiple strokes and bilateral carotid dissections: A fulminant case of newly diagnosed ehlers-danlos syndrome type iv. *Journal of the Neurological Sciences*. 2012;318:168-170
40. Grond-Ginsbach C, De Freitas GR, Campos CR, Thie A, Caso V, MacHetanz J, et al. Familial occurrence of cervical artery dissection - coincidence or sign of familial predisposition? *Cerebrovascular Diseases*. 2012;33:466-470
41. Morren J, Govindarajan R, Caminero AS, Jones D, Rodriguez A, Salgado E. Quadruple extracranial arterial dissection with reversible cerebral vasoconstriction syndrome (rcvs) in a habitual cocaine user presenting with thunderclap headache.

Neurology. Conference: 64th American Academy of Neurology Annual Meeting. New Orleans, LA United States. Conference Start. 2012;78
42. Allam H, Alawi A, Alshekhlee A, Vora N, Mehta S. Bilateral carotid and vertebral artery dissection following cesarean section. *Neurology. Conference: 65th American Academy of Neurology Annual Meeting. San Diego, CA United States. Conference Start*. 2013;80
43. Mawet J, Boukobza M, Franc J, Sarov M, Bousser MG, Ducros A. Reversible cerebral vasoconstriction syndrome and cervical artery dissection in 20 patients. *Cephalalgia*. 2013;33:270-271
44. Panicio MI, Foresto RD, Mateus L, Monzillo PH, Alves MB, Silva GS. Pneumococcal meningitis, cerebral venous thrombosis, and cervical arterial dissection: A run of bad luck? *Neurohospitalist*. 2013;3:20-23
45. Aronov M, Shevchenko NS, Amosova NA, Kotenko KV. Acute three-vessel cervical arterial occlusion due to spontaneous quadruple cervical artery dissection. *BMJ Case Reports*. 2014;(no pagination)
46. Goyal N, Male S, Doss VT, Elijovich L. Spontaneous dissection of bilateral carotid and vertebral arteries with successful endovascular treatment. *Journal of Neuroimaging*. 2014;24 (3):311
47. Vela-Duarte D, Gill R, Pasquale D, Flaster M, Biller J. Multiple spontaneous dissections of cervicocephalic arteries after yoga. *Annals of Neurology*. 2014;76:S89
48. Durand-Dubief F, Marignier R, Benoit A, Roggerone S, Gosset-Janin C, Ionescu I, et al. Ischemic stroke with spontaneous multiple cervical artery dissections in a 40-y old woman during a first course of alemtuzumab: Cause or coincidence? *Multiple Sclerosis*. 2015;1):699
49. Melikyan G, Kamran S, Akhtar N, Deleu D, Miyares FR. Cortex-sparing infarction in triple cervical artery dissection following chiropractic neck manipulation. *Qatar Medical Journal*. 2015
50. Abe A, Nito C, Sakamoto Y, Nogami A, Hokama H, Takahashi S, et al. Spontaneous bilateral cervical internal carotid and vertebral artery dissection in a japanese patient without collagen vascular disease with special reference to single-nucleotide polymorphisms. *Journal of Stroke and Cerebrovascular Diseases*. 2016;25:e114-e117
51. Vannucchi V, Masotti L, Moroni F, Scerra C, Puliti S, Seravalle C, et al. A rare case of contemporary bilateral carotid and right vertebral spontaneous dissection. *Italian Journal of Medicine*. 2016;10 (Supplement 2):117
52. Akamatsu Y, Sato K, Endo H, Matsumoto Y, Tominaga T. Ruptured vertebral artery dissecting aneurysm concurrent with spontaneous cervical internal carotid artery dissection: A report of three cases and literature review. *World Neurosurgery*. 2017;107:1048.e1041-1048.e1046
