# Supplementary material for: Triple and quadruple cervical artery dissections: a systematic review of individual patient data
Source: J Neurol. 2019 Mar 23;266(6):1383–8. doi: 10.1007/s00415-019-09269-1 (PMC6517349; doi:10.1007/s00415-019-09269-1)
Supplement: Supplementary file 3 — Supplementary material 3 (PDF 27 KB) [file 415_2019_9269_MOESM3_ESM.pdf]

Online Resource

Triple and quadruple cervical artery dissections: a systematic review of individual patient data
Journal of Neurology

Valeria Guglielmi, Jeldican Visser, Marcel Arnold, Hakan Sarikaya, René van den Berg, Paul J. Nederkoorn, Didier Leys, David Calvet, Manja Kloss, Alessandro Pezzini, Turgut Tatlisumak, Sabrina Schilling, Stéphanie Debette, Jonathan M. Coutinho
Correspondence to: Jonathan M. Coutinho, Department of Neurology, Amsterdam UMC, location AMC, Amsterdam, The Netherlands. E-mail: j.coutinho@amc.nl

Table II Clinical characteristics, underlying disease/triggering events and outcome in current study, compared to data from CADISP study[1]

	n/N (%)			
	Current study	CADISP study[1]		CADISP study[1]
	Triple/quadruple CeAD (N=96)	Single CeAD (N=833)	P-value[a]	Double CeAD (N=130)[b]
Demographics				
Mean age, y (SD)[c]	42 (9)	44 (10)	0.063	43 (10)
Sex (% women)	63/96 (66%)	351/833 (42%)	<0.001	65/130 (50%)
Medical History				
Smoking	20/72 (28%)	238/822 (29%)	0.833	29/129 (22%)
Hypertension	16/72 (22%)	206/824 (25%)	0.601	38/129 (29%)
Migraine	25/72 (35%)	302/820 (37%)	0.722	51/128 (40%)
Diabetes	4/72 (6%)	16/827 (2%)	0.137	4/129 (3%)

Hypercholesterolemia	12/72 (17%)	161/818 (20%)	0.535	19/125 (15%)
Clinical characteristics				
Headache	63/92 (69%)	542/807 (67%)	0.799	84/128 (66%)
Neck pain	40/92 (44%)	380/807 (47%)	0.511	76/128 (59%)
Horner syndrome	31/92 (34%)	237/807 (29%)	0.390	27/128 (21%)
TIA	17/92 (19%)	175/833 (21%)	0.570	20/130 (15%)
Cerebral infarct	52/91 (57%)	547/833 (66%)	0.106	87/130 (67%)
Underlying disease				
Fibromuscular dysplasia	12/74 (16%)	23/611 (4%)	<0.001	14/105 (13%)
Hereditary connective tissue disorder	4/50 (8%)	3/808 (0.4%)	<0.001	0/128 (0%)
Triggering events				
Recent infection (previous week)	16/90 (18%)	149/813 (18%)	0.898	33/128 (26%)
Recent trauma (previous month)	32/90 (35%)	325/817 (40%)	0.436	57/129 (44%)
Prior cervical manipulation	12/90 (13%)	49/817 (6%)	0.008	15/129 (12%)
Outcome				
mRS 0-2[d]	65/74 (88%)	702/797 (88%)	0.951	110/125 (88%)
Recurrence of CeAD	2/80 (3%)	18/802 (2%)	1.000	4/127 (3%)

[a] Statistical testing performed for comparison between triple/quadruple CeAD patients from current study and single CeAD patients from CADISP study.
[b] Of 149 patients with multiple CeAD in CADISP study, 19 patients had triple or quadruple CeAD, resulting in 130 with double dissection.
[c] Calculated from data of 96 patients (current study), 964 patients (CADISP).
[d] Time to last clinical follow-up: current study median 4 months (range 0-98), CADISP 3 months.
CeAD, cervical artery dissection.

References in Table
1. Béjot Y, Aboa-Aboulé C, Debette S, *et al.* Characteristics and outcomes of patients with multiple cervical artery dissection. *Stroke* 2014;45:37-41.
